# Supplementary material for: Skyrmion bag robustness in plasmonic bilayer and trilayer moiré superlattices
Source: Nanophotonics. 2025 Mar 10;14(23):3955–64. doi: 10.1515/nanoph-2024-0581 (PMC12617697; doi:10.1515/nanoph-2024-0581)
Supplement: Supplementary file 1 — Supplementary Material Details [file j_nanoph-2024-0581_suppl_001.pdf]

Skyrmion Bag Robustness in Plasmonic Bilayer and Trilayer Moiré Superlattices: Supplementary Information

Julian Schwab[1], Florian Mangold[1], Bettina Frank[1], Timothy J. Davis[1,2,3], Harald Giessen[1]*

[1]4th Physics Institute, Research Center SCoPE, and Integrated Quantum Science and Technology Center, University of Stuttgart; 70569 Stuttgart, Germany.

[2]Faculty of Physics and Center for Nanointegration, Duisburg-Essen (CENIDE). University of Duisburg-Essen; 47048 Duisburg, Germany.

[3]School of Physics, University of Melbourne; Parkville Victoria 3010, Australia.

**Corresponding author. Email: giessen@pi4.uni-stuttgart.de*

Commensurability of bilayer and trilayer moiré superlattices

In a hexagonal lattice, all points of the same kind in the lattice can be reached by a translation along $m\boldsymbol{a_1} + n\boldsymbol{a_2}$, where m and n are integers and $\boldsymbol{a_1} = a\left(1/2, \sqrt{3}/2\right)^{\boldsymbol{T}}$ and $\boldsymbol{a_2} = a\left(-1/2, \sqrt{3}/2\right)^{\boldsymbol{T}}$ are the lattice vectors with lattice constant $a = 2\,\lambda_{\mathrm{SPP}}/\sqrt{3} \approx 0.9$ µm. In twisted bilayer graphene, periodic superlattices are achieved by rotating one layer of graphene over another layer, so that two atomic sites overlap:

$$k\boldsymbol{a_1} + l\boldsymbol{a}_2 \rightarrow m\boldsymbol{a_1} + n\boldsymbol{a_2}, \qquad k, l, m, n \in \mathbb{Z}. \tag{S1}$$

Solutions of all commensurate structures have been derived in Ref. [1–5]. Here, we apply these solutions to moiré skyrmion lattices. Following Ref. [4], twist angles of commensurate structures with $0 < \phi < \pi/3$ are given by

$$\cos\phi(m,r) = \frac{3m^2 + 3mr + r^2/2}{3m^2 + 3mr + r^2}, \tag{S2}$$

where $r = n - m$ and m are coprime positive integers. That is, the twist corresponds to the rotation of the lattice site at $n\boldsymbol{a_1} + m\boldsymbol{a_2}$ to the lattice site $m\boldsymbol{a_1} + n\boldsymbol{a_2}$ (see eq. 1), as depicted in Fig. 2a for $n = 3$ and $m = 2$. Thereby it is important to note the symmetry of the twist angle around 30° and all other axes reached by additional rotations of 60°. The primitive vectors of the resultant superlattice (see Fig. 2) are given by

$$\begin{bmatrix}\boldsymbol{t_1}\\ \boldsymbol{t_2}\end{bmatrix} = \boldsymbol{M}(m,r)\begin{bmatrix}\boldsymbol{a_1}\\ \boldsymbol{a_2}\end{bmatrix}, \text{ where} \tag{S3}$$

$$\boldsymbol{M}(m,r) = \begin{cases}\begin{bmatrix} m & m+r \\ -(m+r) & 2m+r\end{bmatrix}, & \text{if } \gcd(r,3) = 1 \\ \begin{bmatrix} m + r/3 & r/3 \\ -r/3 & m + 2r/3\end{bmatrix}, & \text{if } \gcd(r,3) = 3\end{cases} \tag{S4}$$

with the greatest common divisor gcd. The moiré periodicity is defined by $L = a/[2\sin(\phi/2)]$ and equals the length of the superlattice vectors if $r = 1$. In this case the moiré period falls onto a lattice site and the lattice can repeat itself within a minimum distance $||\boldsymbol{t_{1,2}}|| = L$. This property simplifies the observation of multiple moiré unit cells within a small section of the lattice.

In trilayer moiré superlattices periodicity is achieved by a commensurate interference of the moiré periodicity between L1 and L2 with the moiré lattice created by L1 and L3, or L2 and L3, respectively.

This means that the TMSL is periodic if two twist angles out of $\{\phi_{12}, \phi_{23}, \phi_{13}\}$ are chosen to be bilayer commensurate angles that fulfill equation S2.

When ϕ_{12} and ϕ_{23} are commensurate, the periodic superlattice between L1 and L2 is modulated by ϕ_{23}. We assume a virtual fourth layer that is twisted by $\phi_{34} = \phi_{12}$. This creates another superlattice between L3 and L4 that is identical to the superlattice between L1 and L2. The two superlattices are twisted relative to each other by ϕ_{13} and form a superordinate lattice that becomes periodic with the mapping

$$k\boldsymbol{t_1} + l\boldsymbol{t}_2 \rightarrow m\boldsymbol{t_1} + n\boldsymbol{t_2}, \qquad k, l, m, n \in \mathbb{Z}. \tag{S6}$$

Since this equation yields the same results as eq. S1, the four-layer – and therefore also the trilayer moiré superlattice - are periodic if ϕ_{13} fulfills eq. S3. Consequently, the trilayer superlattice vectors are given by

$$\begin{bmatrix} \boldsymbol{t_1} \\ \boldsymbol{t_2} \end{bmatrix} = \boldsymbol{M}(m_2, r_2)\, \boldsymbol{M}(m_1, r_1) \begin{bmatrix} \boldsymbol{a_1} \\ \boldsymbol{a_2} \end{bmatrix}, \tag{S5}$$

where (m_1, r_1) and (m_2, r_2) correspond to ϕ_{12} and ϕ_{13}, respectively. In such a periodic trilayer lattice, all three layers have a lattice site at the center of the super unit cells. Therefore, also L2 and L3 constitute a periodic bilayer superlattice, but this one is created with an additional rotation of L2 by ϕ_{12}, which means that ϕ_{23} does not need to be a bilayer commensurate angle. The reasoning can also be applied when the two other twist angle pairs are chosen to be bilayer commensurate angles. By inserting the virtual fourth layer between L1 and L2, the two superlattices form between L1 and L4, as well as L2 and L3 with $\phi_{14} = \phi_{23}$ and a relative twist between the two superlattices of ϕ_{12}. Therefore, the trilayer superlattice also becomes periodic when ϕ_{23} and ϕ_{12} are bilayer commensurate angles. Analogously, the addition of the fourth layer before L1 forms two superlattices between L4 and L1, as well as L2 and L3 with twist angle $\phi_{41} = \phi_{23}$. In this case, the trilayer superlattice is periodic by using the bilayer commensurate twist angle pair ϕ_{23} and ϕ_{13}.

Surface plasmon waves in the plane wave approximation

In the limit of an infinitely long boundary line that is excited using a continuous wave laser source, the electric field of the launched SPP wave at the perpendicular distance r to the boundary is given by

$$\boldsymbol{E}_{\mathbf{SPP}}(r, t) \approx E_L(\hat{\boldsymbol{e}} \cdot \hat{\boldsymbol{n}}) \left(\frac{i\gamma\hat{\boldsymbol{n}} - \alpha\hat{\boldsymbol{z}}}{k_0} \right) e^{i(\alpha r - \omega_0 t)}, \tag{S1}$$

where $\hat{\boldsymbol{n}}$ is the unit normal vector of the boundary and $\hat{\boldsymbol{e}}$ is the polarization vector of the incident light [6]. α_{SPP} is the in-plane and $\gamma = \sqrt{\alpha^2 - k_0^2}$ the out-of-plane component of the SPP electric field wave number with the vacuum wave number $k_0 = 2\pi/\lambda_0$ of the excitation laser with wavelength λ_0 and angular frequency ω_0. In the plane wave approximation, damping of the plasmon wave is neglected and the in-plane wave vector is purely real: $\alpha_{SPP} = 2\pi/\lambda_{SPP}$. Additionally, it is assumed that the SPP waves propagate through other boundaries without being reflected or losing energy by radiating light. The electric field distribution of structures with multiple boundaries is then simply calculated by summing up the electric field of the plasmon wave of each boundary. This ideal approximation is used for the electric field distributions of Fig. 2 and 3.

Details on disturbance fields

We set the disturbance field to consist of N SPP plane waves in the form of

$$\boldsymbol{E_V}(r) = \sum_{j=0}^{N} E_j \frac{i\gamma_j \boldsymbol{\hat{n}_j} - \alpha_j \boldsymbol{\hat{z}}}{k_0} G_a(r) e^{i(\alpha_j r + \phi_j)},$$

where r is the distance along the propagation direction and $G_a(r)$ adds a centered Gaussian pulse shape with temporal width Δt_{SPP}.

$$G_a(r) = \exp\left(-\frac{4\,Re(\alpha_j) r}{\omega_0^2 \Delta t_{SPP}^2}\right).$$

In our test, we choose $N = 50$ waves with randomly distributed amplitude E_j, phase ϕ_j, propagation constant α_j, and propagation direction $\boldsymbol{\hat{n}_j}$. E_j is chosen to be normally distributed with μ = 1 and σ = 1 and α_j is chosen to be normally distributed with $\mu = \alpha_{SPP}$ and $\sigma = \alpha_{SPP}/10$. The distribution of the propagation constant α_j of the SPP waves must be rather narrow around α_{SPP}, since perturbating waves with other propagation constants could be easily removed via Fourier filtering. The angle of the in-plane propagation direction $\boldsymbol{\hat{n}_j}$ and the phase ϕ_j are uniformly distributed within the half-open interval [0, 2π). Here we assume an excitation of these perturbating waves from the same light source, so that they all have the same frequency ω_0. In this case, the confinement of the waves γ_j is calculated using $\gamma_j = \sqrt{\alpha_j^2 - k_0^2}$.

Robustness of skyrmion bags with different size

We classify the topology of a disturbed field as correct, if the topological charges of the skyrmion bag fulfill $0.97 < S_{bag}/(N-1) < 1.01$ and $0.97 < S_{cluster}/N < 1.01$. In case of the skyrmion lattice (see Fig. 5b and c) the topology is classified as correct if the seven skyrmions in the center of the skyrmion lattice all fulfill $0.97 < S < 1.03$.

In the following we present the results of the analysis of the skyrmion bag robustness for skyrmion bags harboring 1, 2, 3, 4, 9, 10, 12, 14, and 19 skyrmions. The resulting optimal twist angles are listed in Table S1.

N	1	2	3	4	7	10	12	14	19
Bilayer, plane wave	30°	24.5°	21.375°	18.75°	14.75°	13.25°	11.7°	10.4°	9.25°
Bilayer, Huygens	30°	24.5°	22.6°	20.3°	16.7°	14.65°	13°	11.375°	9.7°
Trlayer, plane wave	20°	18.5°	15°	13.125	9.75°	8.7°	7.6°	6.75°	6°
Trilayer, Huygens	20°	19.5°	15.75°	14.25°	9.125°	7.7°	6.5°	5.65°	4.9°

Tab. S1. Ideal twist angles for bilayer and trilayer moiré skyrmion lattices that create skyrmion bags of size N. The results are obtained from the calculations presented in Fig. S1-S3.

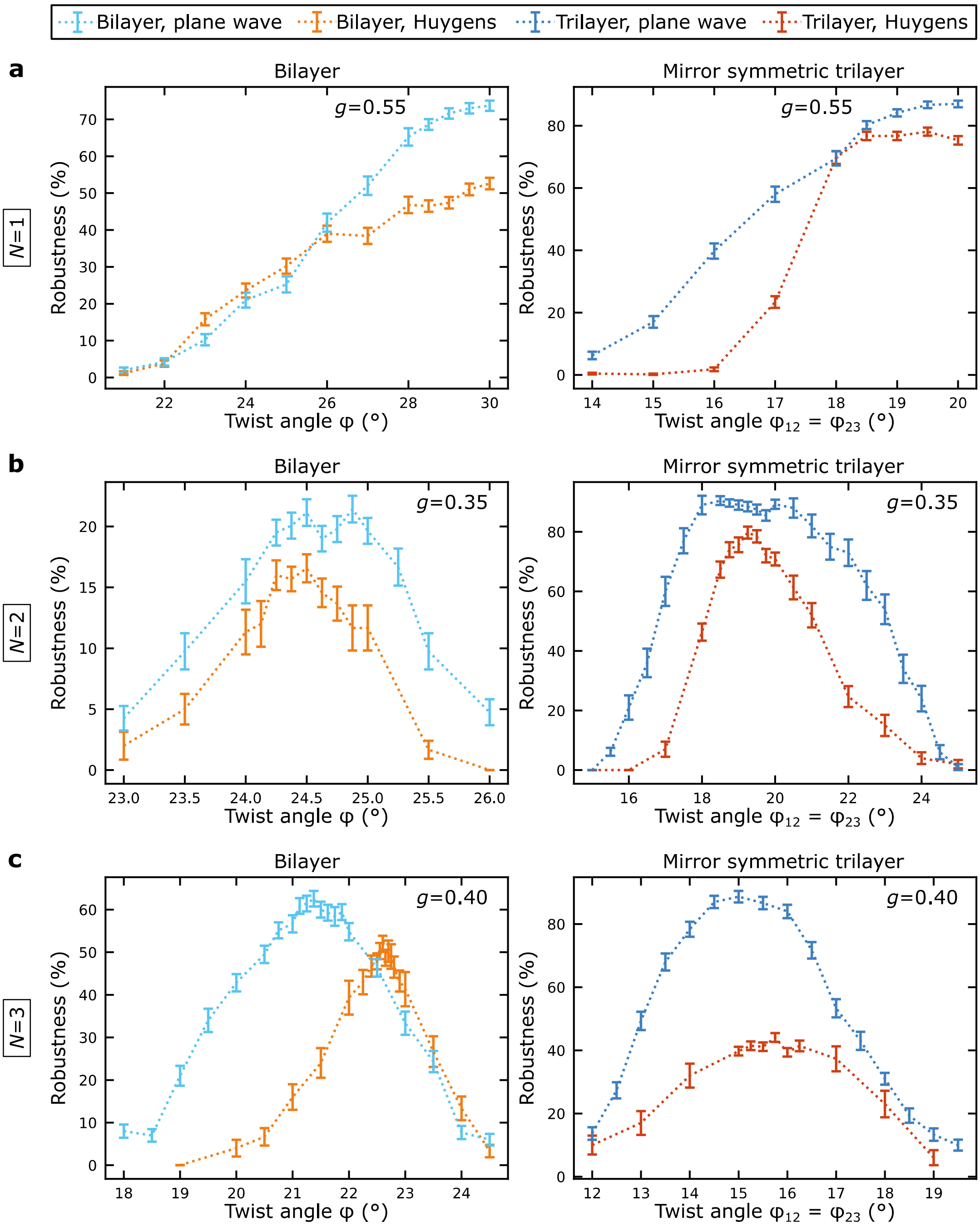

Figure S1 | Robustness of skyrmion bags with $N = 1, 2, 3$ as a function of the twist angles. The results are presented for bilayer and trilayer structures, as well as initial fields that are calculated using the plane wave and the Huygens method. The strength of the perturbational fields are given by g.

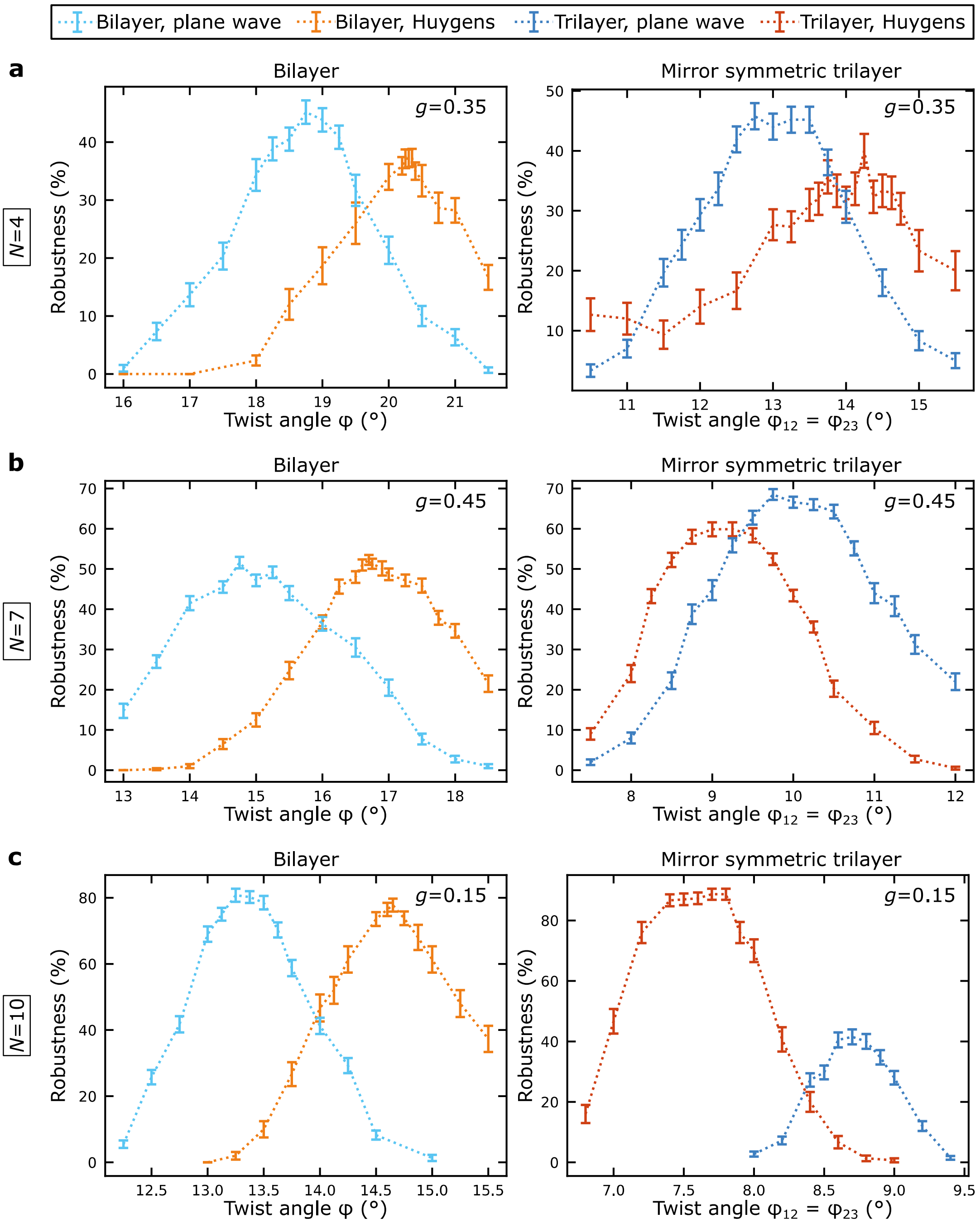

Figure S2 | Robustness of skyrmion bags with $N = 4, 7, 10$ as a function of the twist angles. The results are presented for bilayer and trilayer structures, as well as initial fields that are calculated using the plane wave and the Huygens method. The strength of the perturbational fields are given by g.

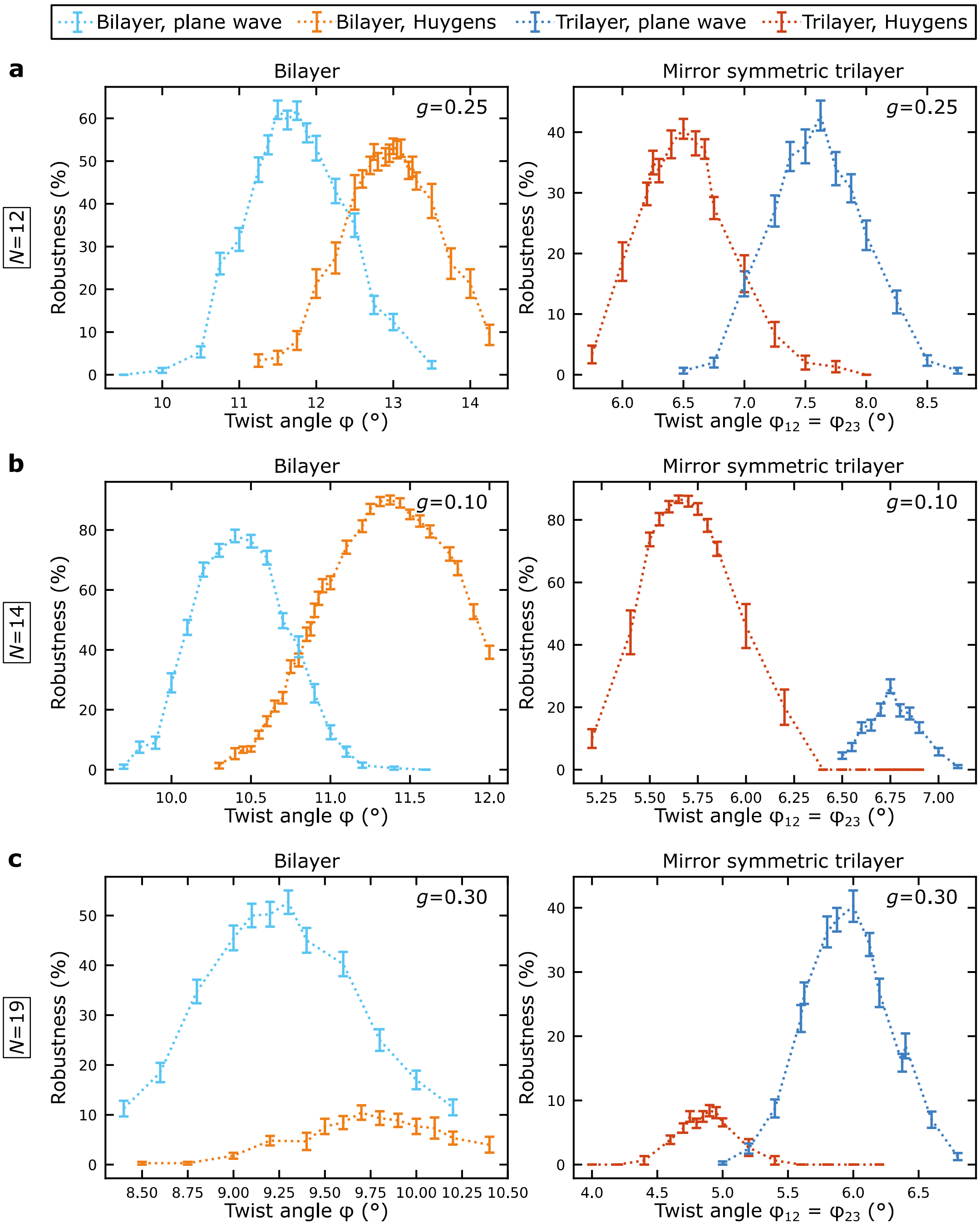

Figure S3 | Robustness of skyrmion bags with $N = 12, 14, 19$ as a function of the twist angles. The results are presented for bilayer and trilayer structures, as well as initial fields that are calculated using the plane wave and the Huygens method. The strength of the perturbational fields are given by g.

References

[1] P. Zeller and S. Günther, 'What are the possible moiré patterns of graphene on hexagonally packed surfaces? Universal solution for hexagonal coincidence lattices, derived by a geometric construction', *New J Phys*, vol. 16, no. 8, p. 083028, Aug. 2014, doi: 10.1088/1367-2630/16/8/083028.

[2] S. Shallcross, S. Sharma, E. Kandelaki, and O. A. Pankratov, 'Electronic structure of turbostratic graphene', *Phys Rev B Condens Matter Mater Phys*, vol. 81, no. 16, p. 165105, Apr. 2010, doi: 10.1103/PHYSREVB.81.165105/FIGURES/10/MEDIUM.

[3] M. Le Ster, T. Märkl, and S. A. Brown, 'Moiré patterns: a simple analytical model', *2d Mater*, vol. 7, no. 1, p. 011005, Nov. 2019, doi: 10.1088/2053-1583/AB5470.

[4] J. M. B. L. dos Santos, N. M. R. Peres, and A. H. C. Neto, 'Continuum model of the twisted graphene bilayer', *Phys Rev B*, vol. 86, no. 15, p. 155449, Oct. 2012, doi: 10.1103/PhysRevB.86.155449.

[5] J. M. Campanera, G. Savini, I. Suarez-Martinez, and M. I. Heggie, 'Density functional calculations on the intricacies of Moiré patterns on graphite', *Phys Rev B Condens Matter Mater Phys*, vol. 75, no. 23, p. 235449, Jun. 2007, doi: 10.1103/PHYSREVB.75.235449.

[6] T. J. Davis, B. Frank, D. Podbiel, P. Kahl, F. J. Meyer Zu Heringdorf, and H. Giessen, 'Subfemtosecond and Nanometer Plasmon Dynamics with Photoelectron Microscopy: Theory and Efficient Simulations', *ACS Photonics*, vol. 4, no. 10, pp. 2461–2469, Oct. 2017, doi: 10.1021/ACSPHOTONICS.7B00676.
